# Morphological Characterization of Plasma-Derived Nanoparticles Isolated by High-Speed Ultracentrifugation: A Scanning Electron Microscopy Study

**DOI:** 10.3390/ijms26199422

**Published:** 2025-09-26

**Authors:** Lubov A. Kungurova, Alexander A. Artamonov, Evgeniy A. Grigoryev, Aleksei Yu. Aronov, Olga S. Vezo, Ruslan I. Glushakov, Kirill A. Kondratov

**Affiliations:** 1 S. M. Kirov Military Medical Academy, St. Petersburg 194044, Russia; liubove.kungurova.lk@gmail.com (L.A.K.); artamonov.alandr@yandex.ru (A.A.A.);; 2 Saint-Petersburg State University, St. Petersburg 199034, Russia; 3 Institute of Experimental Medicine, St. Petersburg 197022, Russia

**Keywords:** exomeres, supermeres, low-voltage scanning electron microscopy, blood plasma fractionation, differential ultracentrifugation, extracellular particles, dynamic light scattering

## Abstract

Extracellular vesicles are critical mediators of intercellular signaling. Recent studies have revealed that, in addition to vesicular structures, smaller non-vesicular nanoparticles—termed exomeres and supermeres—also participate in intercellular communication. Detailed characterization of these nanoscale entities within biological systems is essential for elucidating their structural and functional roles. Due to their sub-50 nm dimensions, high-resolution imaging modalities such as atomic force microscopy and electron microscopy are currently the primary techniques available for their visualization. In the present study, we employed low-voltage scanning electron microscopy to investigate the size of exomeres and supermeres isolated from human blood plasma via high-speed ultracentrifugation. Platelet-poor plasma was obtained from the blood of six healthy donors (two women and four men, aged 21–46 years). By ultracentrifugation (170,000× *g* for 4 h), the plasma was purified of extracellular vesicles. Two fractions were sequentially isolated: one containing exomeres (170,000× *g* for 20 h) and one containing supermeres (370,000× *g* for 20 h). The particles were examined using a Zeiss Auriga microscope with no sputter coating at an accelerating voltage of 0.4–0.5 kV. The images obtained from the fractions showed particles 10–50 nm in diameter, both individual particles and aggregated structures. The fractions were also slightly contaminated with larger particles, supposedly extracellular vesicles. Examining the fractions using a dynamic light scattering device additionally revealed the presence of particles 10–18 nm in size. It should be noted that the fractions obtained did indeed contain particles measuring 10–50 nm, which corresponds to the size of exomeres and supermeres. Low-voltage scanning electron microscopy allows for examination of the structure of exomeres and supermeres in blood plasma fractions. However, it should be noted that without the use of immunological identification, this method does not allow exomeres and supermeres to be distinguished from accompanying particles. It should also be noted that because the size of exomeres and supermeres is close to the detection threshold of low-voltage scanning electron microscopy, in such studies it is generally only possible to detect the size of these particles.

## 1. Introduction

Traditionally, single protein molecules, cholesterol derivatives, or certain other low-molecular-weight compounds were considered to be the primary agents of signal transmission between cells. However, over the past decade, it has been demonstrated that signals are transmitted not only by low-molecular-weight compounds but also by more complex structures, namely extracellular vesicles (EVs). It is known that these particles carry a wide range of different biomolecules (lipids, proteins, RNA) [1]. It has recently been shown that smaller, presumably non-membrane structures—exomeres and supermeres—can transmit intercellular signals and transport proteins and RNA [2]. In particular, it has been shown that exomeres are capable of healing wounds by altering inflammatory processes through their effects on the FOXE and PIP3-AKT signaling pathways [3], while tumor cell supermeres reduce lipid and glycogen levels in the liver and increase lactate secretion and resistance to cetuximab [2]. For these reasons, these structures are not only important as promising therapeutic agents, but also as possible markers for various diseases. The great importance of these structures necessitates the development of methods for studying the morphology of exomeres and supermeres and assessing their sizes. The asymmetric flow field–flow fractionation was initially used to isolate these particles [4]. This technique allows for the production of high-purity particle sample preparations; however, the volume of exomeres and supermeres samples is relatively small. To obtain large volumes, a method of differential ultracentrifugation was proposed, with preliminary removal of albumins by sorption and vesicles by ultracentrifugation, followed by precipitation of exomeres and supermeres [2]. It has been shown that with such ultracentrifugation, it is possible to separate most of the EVs from exomeres and to separate exomeres and supermeres from each other.

Several specific methods are used to estimate the size of EVs [5]. These include nanoparticle tracking analysis (NTA), tunable resistive pulse sensing (TRPS), and dynamic light scattering (DLS). However, it should be noted that NTA and TRPS are not well suited for such small particles, as they cannot detect particles smaller than 30 nm. For this reason, the DLS method is most suitable for assessing the size of such small particles (less than 30 nm).

Previously, the morphology of supermeres was studied using transmission electron microscopy [6] and atomic force microscopy [2]. However, no studies of these particles have been conducted using low-voltage scanning electron microscopy (LVSEM) [7]. Nevertheless, this method has a number of advantages over other types of microscopy. LVSEM allows direct examination of the surface of particles. Such images cannot be obtained using transmission electron microscopy with negative contrast, for example, with uranyl acetate, because in this case it is not the surface of the particle itself that is fixed, but essentially its imprint in electron-dense metal. Nor can it be obtained using traditional SEM, since this method involves sputtering a layer of metal, usually gold, onto the sample to reflect electrons from the sample surface. This layer, which is several nanometers thick, significantly distorts such small particles (less than 50 nm) as exomeres and supermeres. Cryo-electron microscopy, although it allows the construction of layer-by-layer three-dimensional models of extremely small particles, also does not allow direct examination of the sample surface. Such examination is possible with atomic force microscopy (AFM) and LVSEM. However, it is LVSEM that allows us to quickly obtain images with a large field of view and greater depth of field. Previously, our research group used this method to study the morphology of EVs obtained from blood plasma [8]. However, the surface morphology of exomeres and supermeres using LVSEM has not yet been studied. The objectives of our research were to study the morphology of exomeres and supermeres obtained by differential centrifugation using low-voltage scanning electron microscopy, to evaluate and compare their sizes by SEM imaging and dynamic light scattering.

## 2. Results

**Figure 1:**
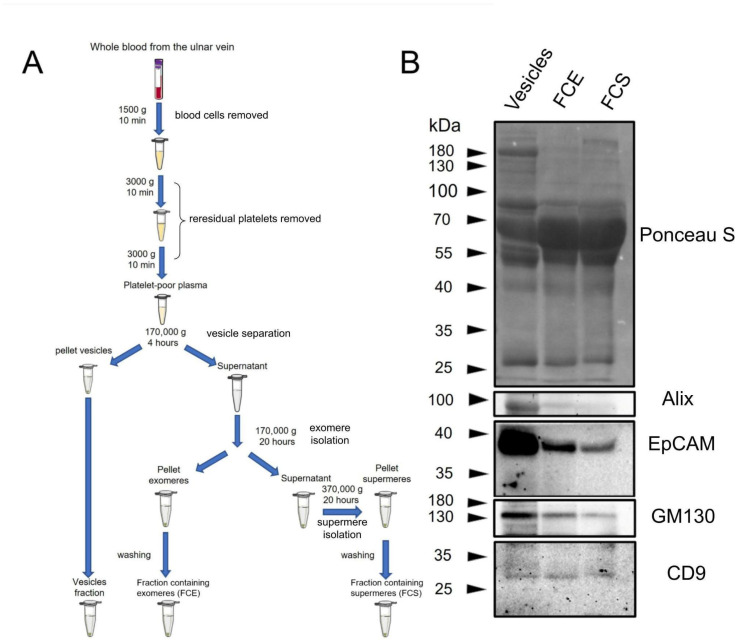
(**A**) Schematic representation of the isolation of fractions containing exomeres (FCE) and supermeres (FCS). (**B**) Immunoblot analysis of extracellular vesicles, FCE, and FCS samples with Alix, EpCAM, ICAM, and CD9 protein antibodies.

**Figure 2:**
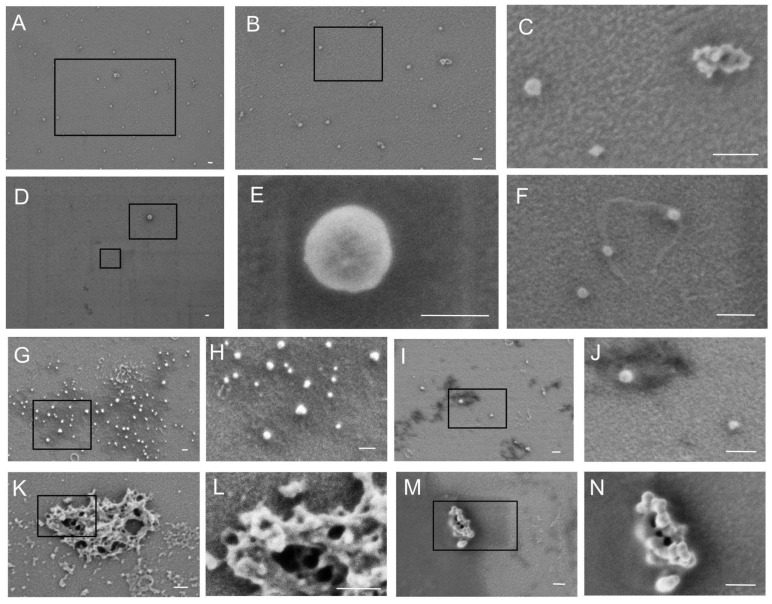
Low-voltage scanning electron micrographs of particles in the FCE fraction. (**A**,**B**,**D**,**G**,**I**,**K**,**M**) Overview images. (**B**,**C**,**E**,**F**,**H**,**J**,**L**,**N**) Detailed images. (**E**) Individual particle on a light substrate. (**A**–**D**) Field of particles on a light substrate. (**I**,**J**) Particles located near the border of light and dark substrate regions. (**G**,**H**) Field of particles on a dark substrate. (**C**,**K**–**L**) Aggregates of particles on a light substrate. (**M**,**N**) Aggregates of particles located near the border of light and dark substrate regions. The length of the white scale bar corresponds to 100 nm.

**Figure 3:**
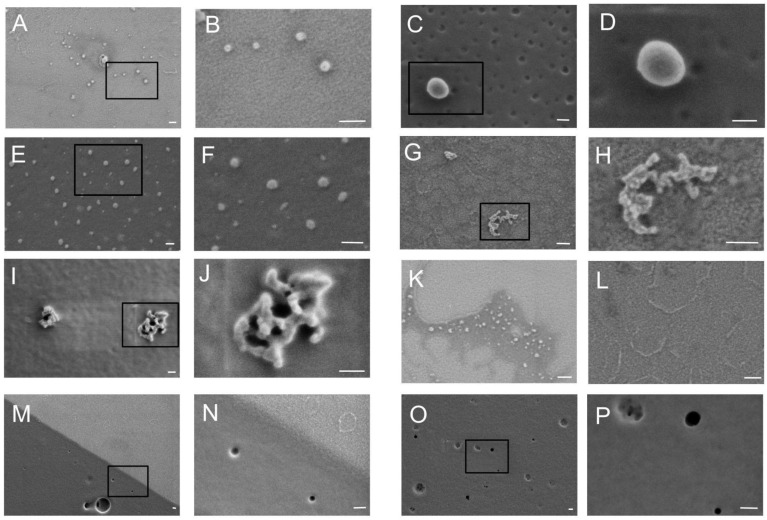
Low-voltage scanning electron micrographs of particles in the FCS fraction, PBS and serum albumin. (**A**,**C**,**E**,**G**,**I**,**M**,**O**) Overview images. (**B**,**D**,**F**,**H**,**J**,**K**,**L**,**N**,**P**) Detailed images. (**A**–**F**) Individual particles of varying sizes. (**A**,**B**) Field of particles on a light substrate. (**E**,**F**) Field of particles on a dark substrate. (**K**) Particles located near the border of light and dark substrate regions. (**G**–**J**) Aggregates of particles. (**L**) PBS control. (**M**–**P**) Serum albumin control. (**M**,**N**) The boundary between the dark and light surfaces in the albumin control. (**O**,**P**) Dark surface in the albumin control. The length of the white scale bar corresponds to 100 nm.

Initially, we compared the overall protein profile in FCE, FCS, and vesicle fractions using non-specific Ponceau S membrane staining (Figure 1B). As can be seen in the figure, the protein profile in different fractions does not differ significantly. The vesicle fraction contains significantly less of the major 65 kDa protein. This fraction also contains a 180 kDa protein that is virtually absent in other fractions. In FCS, there is a protein of about 200 kDa, which is practically absent in FCE and vesicles. Since we did not perform mass spectrometry analysis, we do not know which proteins correspond to these bands. Then, vesicle markers were detected in FCE, FCS, and vesicle fractions using immunoblotting. (Figure 1B). According to the immunoblotting results, the amount of Alix, EpCAM, and GM130 proteins was significantly higher in vesicle fractions compared to FCE and FCS. Both FCE and FCS contained small but detectable amounts of Alix, EpCAM, and GM130. The levels of these proteins were slightly lower in FCS than in FCE. The CD9 protein was distributed at virtually the same level between the three fractions. The subsequent stage involved scanning electron microscopy. Micrographs of the prepared samples are presented in Figure 2A–K and Figure 3A–K. The PBS control is shown in Figure 3L. The albumin control is shown in Figure 3M–P. The FCE (Figure 2) and FCS (Figure 3) samples did not differ significantly. The background of the samples in both cases was either light with a grainy pattern (Figure 2A–F,K,L and Figure 3A,B,G,H) or dark with a relatively smooth surface (Figure 2G,H,M,N and Figure 3E,F,I,J). Sometimes the particles were located at the border between the dark and light backgrounds, predominantly on the dark side (Figure 2I,J and Figure 3K). In some cases, the particles lay on a smooth surface with dark holes (Figure 3C,D). In the control sample with pure buffer, the background was always light, sometimes with cord-like structures (Figure 3L). The same structures were found in the FCE samples (Figure 2F,G,I), FCS samples (Figure 3A,G), and in the albumin control (Figure 2M,N). In the FCE and FCS samples, particles were found either solitary (Figure 2A–J and Figure 3A–F,K) or in groups in the form of aggregates (Figure 2K–N and Figure 3G–J). Morphologically, the particles in both fractions were spherical or near spherical in shape.

**Figure 4:**
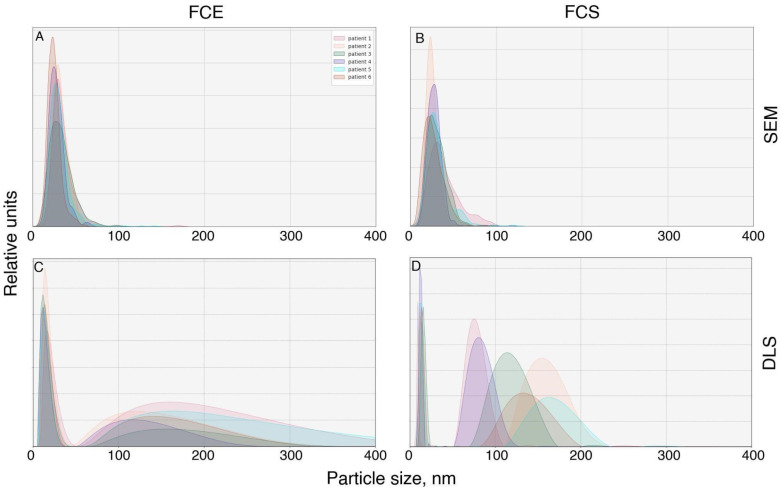
Particle size measurements in FCE (**A**,**C**) and FCS (**B**,**D**) fractions, obtained from micrographs (**A**,**B**) and by dynamic light scattering (**C**,**D**).

We proceeded to assess the particle sizes in FCS and FCE directly from micrographs (Figure 4A,B). Examination of the FCE micrographs revealed that the particle sizes mainly ranged from 9 to 60 nm (Figure 4). Median FCS 27.08+ (st dev) 12.62 nm, Median FCE 29.17+ (st dev) 11.84 nm. Comparison of sizes using the Wilcoxon–Mann–Whitney criterion showed that particles in the FCE fraction are larger than those in the FCS fraction (*p* = 2.2 × 10^−16^).

The DLS study demonstrated a different distribution of particles by size (Figure 4C,D). The majority of particles in FCE and FCS were approximately the same size (6–44 nm for FCE; 8–20 nm for FCS) with modal values of 11–18 nm and 10–15 nm, respectively. However, subpopulations of larger sizes were also detected. The FCE samples obtained from all donors contained particles ranging from 52 to 581 nm with a mode of 110–173 nm. In the FCS samples, the size range of large particles was 60–202 nm with a mode ranging from 70 to 173 nm.

## 3. Discussion

Since albumin was not removed from plasma in our work, it was decided to slightly modify the fractionation method compared to the previously described method [9]. It is likely that albumin could have prevented particle precipitation. Therefore, when purifying vesicles, we increased the rotation speed by ~3.5% (167,000× *g* in [9]. versus 170,000× *g* in our work). The same increase in speed was used when isolating the fraction containing exomeres. When isolating the fraction containing supermers, we increased the speed by less than 1% (367,000× *g* in the work [9] versus 370,000× *g* in our work). Such an increase in speed should not significantly affect the composition of the preparations. We also slightly increased the time both when obtaining the fraction containing exomeres and when obtaining the fraction containing supermers (16 h in the work of Zhang et al. [9] versus 20 h in our work).

Virtually any extracellular physiological fluid contains a complex mixture of particles that carry extracellular signals. One of the researchers’ objectives is to effectively separate this mixture into relatively pure fractions. Therefore, one of the main indicators of the obtained samples is their degree of purity. It should be noted that the samples were likely to have been sufficiently purified of EVs. As can be seen in the immunoblots presented, the level of vesicle marker proteins (Alix, EpCAM, GM130) is much higher in the corresponding fraction (Figure 1B). Nevertheless, it is likely that this purification was incomplete and residual amounts of vesicles were present in FCS and FCE. It should be noted that the CD9 protein is distributed almost evenly between the vesicle fractions and FCS, FCE. This corresponds to the literature data, as it has been shown that this protein can remain in non-vesicular fractions [10], including the exomeres fraction [11]. However, this protein has not been previously detected in the supermer fraction. This may be because the number of studies on supermers is still relatively small. Summarizing the immunophenotyping results obtained using immunoblotting, it should be noted that we did not find any significant differences in the expression levels of the specific proteins studied (Alix, EpCAM, GM130, CD9) or in the overall pattern of band distribution when staining with Ponceau S between FCS and FCE.

The morphology of particles in both fractions was relatively similar. The vast majority of particles were spherical or near spherical in shape. No cup-shaped particles or particles with more complex shapes were detected. This may be due to the fact that the size of exomeres and supermeres is close to the resolution limit of low-voltage scanning electron microscopy.

Most of the particles detected in our fractions were smaller than 50 nm (Figure 2A and Figure 3A). It is likely that most of these structures were exomeres or supermeres in the corresponding fraction. In addition, it is highly probable that both high-density and low-density lipoproteins were present in these fractions. However, some near-spherical structures were significantly larger, up to 100 nm (Figure 2E and Figure 3C,D). These structures were likely EVs.

Other possible contaminants are albumin protein molecules [9]. It is likely that this is due to the ability of albumin to form aggregates [12], and such high-molecular-weight complexes precipitate during ultracentrifugation. As mentioned by other researchers, previously, when isolating exomeres and supermeres by ultracentrifugation, plasma albumin was preliminarily removed [9], although there are studies where this preliminary purification prior to ultracentrifugation was not performed [13].

It should be noted that immunological purification from albumin is not an ideal method for isolating exomeres and supermeres, since some fractions of these particles may be lost with albumin, as it has been shown that albumin forms aggregates with nanoparticles [12]. For this reason, we decided not to remove albumin, but instead try to study the morphology of particles in fractions containing this protein. To reduce the albumin concentration, we performed PBS washing after ultracentrifugation of the FCS and FCE fractions. However, it was not possible to purify FCS and FCE very well by ultracentrifugation. When staining the blot with the non-specific PonseauS dye, a major band at 65 kDa was observed, probably corresponding to serum albumin. It should also be mentioned that some of the particles we examined by electron microscopy were located on the surface of a material that was highly electron-absorbent. This material had a dark color, which contrasted sharply with the glass (Figure 2G,H and Figure 3C–F). This is particularly noticeable at the boundary between the dark and light material (Figure 2I,J,M,N and Figure 3K). This coloring is probably caused by albumin cross-linked with glutaraldehyde on the glass surface. A similar dark coating was observed in samples where we fixed albumin on glass (Figure 3M–P) but was not present in samples containing pure buffer (Figure 3L). The holes obtained during fixation and drying of albumin (Figure 3M–P) are also observed in FCS samples (Figure 3C,D). The cord-like structures visible in both fraction samples (Figure 2L and Figure 3A), the buffer sample (Figure 3L) and albumin (Figure 3M,N) are apparently either glass defects or structures that appear during fixation or drying of any of the samples obtained. It is interesting to note that particles settle slightly better on dark surfaces than on light ones. This is probably due to the fact that albumin allows exomeres and supermeres to adhere more firmly to the glass surface. It should also be reiterated that for optimal observation of particles in our fractions, the best dilution was selected for each individual sample before fixation on glass. If the particles we were looking for were really hidden by dark material in the sample, we diluted the sample again two or more times and fixed it on the glass again. This procedure was repeated until the particles were clearly visible under the microscope. With this method of sample preparation, some of the particles are likely to be obscured by protein, but a significant proportion of them are suitable for morphological observation by scanning electron microscopy.

The particle size distribution obtained by DLS was slightly different. The size of the main subpopulation of particles in both FCS and FCE fractions, obtained by dynamic light scattering, ranged from 6 to 50 nm. These were presumably exomeres and supermeres. Larger particles (from 52 to 581 nm for FCE, from 33 to 430 nm for FCS) may have represented their self-aggregates or complexes with albumins, lipoproteins, or vesicles. However, the monomeric albumin itself was probably removed during washing, since there were almost no particles with a size of 7–4 nm (the diameter of albumin) [14]. It is also possible that these were traces of EVs. It should be noted that the peak value obtained by DLS is not suitable for comparing the number of particles of different sizes in a mixture. Larger particles scatter light stronger and, accordingly, disproportionately increase the peak value obtained by DLS. In our case, this means that the peaks of larger particles, which are particularly noticeable in FCS, are actually much lower in concentration than the supermer particles contained in this fraction.

It should be noted that there is a certain difference in size between the particles measured in micrographs and DLS. This is explained by the fact that in the former case, the electron-dense diameter is measured, while in the latter, the hydrodynamic diameter is measured. It is well known that these two metrics can differ significantly [15].

It is noteworthy to mention that we discovered no significant differences between exomeres and supermeres in terms of electron density size (modal value FCE 17–36 nm; FCS 18–32 nm) and hydrodynamic radius (modal value FCE 11–18 nm; FCS 10–15 nm). This is entirely consistent with the published data, which suggests that exomeres are particles measuring approximately 30 nm and supermeres are smaller than 50 nm [11]. Thus, there is virtually no difference in size between these particles. Apparently, the main difference between these particles lies in their sedimentation coefficient during ultracentrifugation. Since the shape and size of the particles do not differ significantly, and the buffer is not very different either, the difference in sedimentation is probably mainly due to their density. However, to confirm this fact, separate studies of exomeres and supermeres need to be conducted.

Thus, scanning electron microscopy allows us to study the morphology of particles in our fractions, but we cannot distinguish whether we are observing an exomer, supermer, vesicle, or lipoprotein. Determining exactly what kind of particle we are dealing with would allow us to perform immunoprecipitation with antibodies against a specific marker (for example, CD9, CD63 for vesicles; ACE2, HSP90, AGO2 for exomeres and supermeres; APO B, APO A for lipoproteins) and secondary antibodies conjugated with colloidal gold particles. Theoretically, this is possible. Such an analysis has been performed, for example, with lampbrush-type chromosomes [16], which are much larger structures than our particles. However, a possible detectable marker must have a number of properties. It must not only be located on the surface of the particle but also have an antigen recognizable by antibodies also located on the surface. It should be noted separately that colloidal gold particles conjugated with secondary antibodies must be as small as possible. A large particle (25 nm) will be the size of an exomer or supermer, which will at least greatly complicate observation.

If we try to compare microscopy methods suitable for studying the morphology of exomeres and supermeres and allowing us to reliably distinguish these particles from vesicles and lipoproteins, then first and foremost these are transmission electron microscopy [6] and atomic force microscopy [2], which have already been used to study exomeres and supermeres. These methods allow immunological staining [17], including vesicles [18], although this has not yet been performed for exomeres and supermeres. Cryo-electron microscopy not only provides high-quality images of vesicles [19] and lipoproteins [20] but also allows the internal contents of protein complexes to be studied at almost atomic levels. Helium ion microscopy should also be noted, as it allows the surface of vesicles [21] to be studied with deep depth of field [22].

Unfortunately, helium ion and cryo-electron microscopy have not yet been used to study particles such as exomeres and supermeres, but have been applied to vesicles [19,21] and lipoproteins [20]. Surface analysis methods (atomic force and helium ion microscopy) without immunostaining only allow the determination of particle size, but cannot show whether it has a membrane like a vesicle, or whether it is an exomer, supermer, or a lipoprotein. Methods that allow the internal structure to be assessed (TEM and Cryo-TEM) without immunostaining should distinguish between membrane structures (vesicles) and non-membrane exomeres or supermeres. To distinguish exomeres and supermeres from lipoproteins, it is advisable to use colloidal gold immunostaining when using these methods.

Summarizing our work, we can say that removing albumin from blood plasma is not a necessary step in obtaining particles after prolonged high-speed ultracentrifugation for visualizing these structures using low-voltage scanning electron microscopy. The particles can be observed without removing albumin.

### Study Limitations

The low-voltage scanning electron microscopy method we used has serious limitations when it comes to studying exomeres and supermeres.

1. Due to the small size of the particles, which is close to the detection threshold, the main parameter that can be evaluated using this method is particle size.
2. Without additional immunological detection, the method does not allow exomeres and supermeres to be distinguished from each other or from accompanying particles such as vesicles or lipoproteins.

## 4. Materials and Methods

### 4.1. Patients, Blood Sampling and Plasma Fractionation

Platelet-poor blood plasma was obtained from six healthy donors (four men and two women) after signing all necessary consent forms. The full set of clinical data of the patients is presented in the Supplementary Materials. Whole blood from the cubital vein was collected in vacuum tubes with EDTA and centrifuged at 1500× *g* for 10 min. The supernatant was collected twice and centrifuged at 3000× *g* for 10 min to obtain platelet-poor plasma. The procedure for obtaining plasma and further ultracentrifugation is shown in Figure 1. The plasma was centrifuged for four hours at 170,000× *g* to remove vesicles. The supernatant was centrifuged again for 20 h at 170,000× *g*. The precipitate was washed by repeated ultracentrifugation for another 20 h at 170,000× *g* to obtain a fraction containing exomeres (FCE). The supernatant was centrifuged again for 20 h at 370,000× *g*. The precipitate was washed by centrifugation with phosphate-buffered saline (PBS) to obtain a fraction containing supermeres (FCS). PBS used for sample dilution and fraction washing prior to use was ultracentrifuged for 20 h at 370,000× *g* to remove all possible foreign particles. The S.M. Kirov Medical Academy Expert Ethics Council authorized the research (protocol No. 287 of 23 January 2024).

### 4.2. Immunoblotting

The total protein content in three fractions (vesicles, FCE, and FCS) was determined using the RC DC™ Protein Assay Kit II (#5000122; Biorad, Hercules, CA, USA). After equalizing the concentrations, the samples were boiled for ten minutes with Laemmli buffer and transferred to a 10% polyacrylamide gel. After gel electrophoresis, the proteins were transferred to a nitrocellulose membrane (1620112; Biorad). The membrane with transferred proteins was stained with Ponceau S (P7170; Sigma, Oakville, ON, Canada) and photographed. After removing the dye, the membrane was blocked with 5% skimmed milk and stained with antibodies against Alix (#2171; Cell Signaling Technology), EpCAM (#2626; Cell Signaling Technology, Danvers, MA, USA), GM130 (#12480; Cell Signaling Technology), and CD9 (#13174; Cell Signaling Technology) proteins. The membrane was subsequently stained with secondary antibodies conjugated to horseradish peroxidase (#7076, #7074; Cell Signaling Technology) and revealed using the SuperSignal™ West Femto Maximum Sensitivity Substrate kit (34095; ThermoFisher Scientific, Waltham, MA, USA) on a Chemidoc (Biorad).

### 4.3. Sample Preparation for Low-Voltage Scanning Electron Microscopy

Samples were prepared according to a previously described method [8] with slight modifications. SuperFrost Plus (Edpredia, Seattle, WA, USA) slides, pretreated with chromic acid and washed with 7X-ES detergent (097667193, MP Biomedicals, Solon, OH, USA) and deionised water, were used to fix the particles. The optimal concentration of exomeres and supermeres in the sample for observation under a SEM was selected experimentally by diluting the PBS sample 2–4 times. To fix 5 μL of the sample, it was mixed on glass with 5% glutaraldehyde (TC558M-100ML, Himedia, Kennett Square, PA, USA) for 30 min in a humid chamber, after which the samples were dehydrated by passing them through alcohol with increasing concentrations (50%, 75%, 95%) and dried. To obtain control samples, 5 µL of PBS and 5 µL of a solution (1.5 g/L) of bovine serum albumin (#5000007; Bio-Rad) were used instead of the sample.

### 4.4. Low-Voltage Scanning Electron Microscopy and Size Particle Measure

Particle morphology was observed on a Zeiss (Oberkochen, Germany) Auriga scanning electron microscope at 30,000–360,000× magnification and an accelerating voltage of 0.400–0.500 kV. The Feret diameter was chosen as the main parameter for particle size evaluation in the micrographs. The size of the nanoparticles was analyzed using ImageJ2 software (version 1.54 k, 12 November 2024). The size was measured only if the particle’s boundaries were clearly visible. For each patient, 420 to 1987 supermeres and 374 to 864 exomeres were counted. All measurements are presented in the Supplementary Materials.

### 4.5. Dynamic Light Scattering

Prior to carrying out the observations, FCE and FCS samples were adjusted to 1.1 mL with PBS, centrifuged at 3000× *g* for 20 min to remove large aggregates, and 500 μL of supernatant was collected. The resulting sample was diluted 3–6 times. Particle size (hydrodynamic diameter) measurement in fractions was performed on a Photocor Complex (Photocor, Moscow, Russia) at a temperature of 20 °C, at an angle of 90°, with a laser wavelength of 445 nm. The observation was carried out for 10 min. The DynaLS program (v2.x) was used to process the results. The measurement results are presented in the Supplementary Materials. Exomeres were counted.

### 4.6. Statistics

The statistical significance of differences in particle sizes was determined using the nonparametric Wilcoxon–Mann–Whitney criterion in R-studio (version 4.4.1). Seaborn and matplotlib modules for Python (version 3.13.3) were used to construct the Kernel Density Estimate plots.

## Data Availability

Data are contained within the article or Supplementary Material.

## Supplementary Materials

The following supporting information can be downloaded at https://www.mdpi.com/article/10.3390/ijms26199422/s1.
