# Supplementary material for: Morphological Characterization of Plasma-Derived Nanoparticles Isolated by High-Speed Ultracentrifugation: A Scanning Electron Microscopy Study
Source: Int J Mol Sci. 2025 Sep 26;26(19):9422. doi: 10.3390/ijms26199422 (PMC12524877; doi:10.3390/ijms26199422)
Supplement: Supplementary file 1 [file ijms-26-09422-s001.zip › supl clin data.pdf]

	Patient 1	Patient 2	Patient 3	Patient 4	Patient 5	Patient 6
Gender	man	man	woman	man	man	woman
Age	43	32	42	38	21	46
WBC^10^9 /L	11.9	9.8	7.2	5.3	7.0	7.2
RBC^10^{12} /L	5.39	6.53	5.53	5.10	5.85	5.4
HGB g/L	153	182	161	154	173	154
HCT %	48.4	57.4	50.9	47.7	52.7	48.8
MCV fL	89.8	87.9	92.0	93.5	90.1	90.4
MCH Pg	28.4	27.9	29.1	30.2	29.6	28.5
MCHC g/L	316	317	316	323	328	316
PLT^10^9 /L	428	384	285	307	269	341
LY %	42.4	29.9	31.1	46.9	38.1	43.6
MO %	10.9	8.8	17.0	11.8	12.0	13.7
GR %	46.7	61.3	51.9	41.3	49.9	42.7
RDWCV %CV	15.3	15.2	13.3	13.4	12.8	14.8
RDWSD fL	55.0	53.4	48.9	50.1	46.1	53.5
PCT %	0.4	0.38	0.24	0.25	0.26	0.3
MPV fL	9.4	10.0	8.5	8.3	9.8	8.7
PDW %	16.0	17.2	17.6	16.9	16.7	17.2
